# Program for expectant and new mothers: a population-based study of participation

**DOI:** 10.1186/1471-2458-11-691

**Published:** 2011-09-06

**Authors:** Marni D Brownell, Mariette Chartier, Wendy Au, Jennifer Schultz

**Affiliations:** 1 Department of Community Health Sciences, Faculty of Medicine, University of Manitoba, 408-727 McDermot Avenue, Winnipeg, Manitoba, R3E 3P5, Canada; 2 Manitoba Centre for Health Policy, University of Manitoba, 408-727 McDermot Avenue, Winnipeg, Manitoba R3E 3P5, Canada

## Abstract

**Background:**

The Manitoba Healthy Baby Program is aimed at promoting pre- and perinatal health and includes two components: 1) prenatal income supplement; 2) community support programs. The goal of this research was to determine the uptake of these components by target groups.

**Methods:**

Data on participation in each of the two program components were linked to data on all hospital births in Manitoba between 2004/05 through 2007/08. Descriptive analyses of participation by maternal characteristics were produced. Logistic regression analyses were conducted to identify factors associated with participation in the two programs. Separate regressions were run for two groups of women giving birth during the study period: 1) total population; 2) those receiving provincial income assistance during the prenatal period.

**Results:**

Almost 30% of women giving birth in Manitoba received the Healthy Baby prenatal income supplement, whereas only 12.6% participated in any community support programs. Over one quarter (26.4%) of pregnant women on income assistance did not apply for and receive the prenatal income supplement, despite all being eligible for it. Furthermore, 77.8% of women on income assistance did not participate in community support programs. Factors associated with both receipt of the prenatal benefit and participation in community support programs included lower SES, receipt of income assistance, obtaining adequate prenatal care, having completed high school and having depressive symptoms. Having more previous births was associated with higher odds of receiving the prenatal benefit, but lower odds of attending community support programs. Being married was associated with lower odds of receiving the prenatal benefit but higher odds of participating in community support programs.

**Conclusions:**

Although uptake of the Healthy Baby program in Manitoba is greater for women in groups at risk for poorer perinatal outcomes, a substantial number of women eligible for this program are not receiving it; efforts to reach these women should be enhanced.

## Background

The time extending from conception to a child's first birthday is a crucial one in terms of child development and life-long health [1-3]. Maternal factors such as stressful life circumstances, low socioeconomic status, poor nutrition and health, and smoking and alcohol/drug use during pregnancy can adversely influence birth outcomes and newborn health [4-10]. In turn, outcomes such as low birth weight, preterm births and intrauterine growth restriction have an impact on neonatal and infant morbidity and mortality [11] as well as longer-term health, cognitive and behavioural problems [12-20].

Fortunately, a great deal is known regarding not only risk factors, but also some of the protective factors associated with perinatal outcomes. Good prenatal nutrition can have a positive impact on birth weight, gestation and intrauterine growth [21] as well as on neurological development [22-24]. Adequate prenatal care can also have a positive impact on perinatal outcomes through medical, nutritional and educational interventions [25]. There is also abundant evidence on the positive effects of breastfeeding on health in infancy and early childhood [26,27]. The type of parenting an infant receives also has a tremendous impact on health and development; warm and responsive parental care is a protective factor in infancy which leads to secure attachments with parents and healthy neurological and psychological development [28].

Based on this evidence, a number of prenatal and early childhood programs have been developed to improve birth and infant outcomes. These programs can improve outcomes by advocating for prenatal care, encouraging cessation of smoking and alcohol use, providing supplemental incomes, promoting breastfeeding and positive parenting practices, and by decreasing stress through the provision of social and emotional support. Nutrition intervention programs and programs offering income supplements have both demonstrated positive effects on birth outcomes [29-34]. There is also evidence that high-risk mothers and their infants, such as those experiencing a high degree of stress or living in low income situations, benefit from social support programs [35].

Success of prenatal and infant programs is at least partially dependent on the uptake of these programs by target groups; if not all of the most vulnerable women and families participate in the programs, then it is difficult to evaluate their success. Evidence from the Sure Start program in the UK suggests that positive benefits of the program are limited to the less socially deprived participants, with some adverse effects evident in the most disadvantaged families [36,37]. Relatively little information is available on uptake of prenatal and infant programs. An evaluation of the Canada Prenatal Nutrition Program claims that the program is effective at reaching "at-risk" populations, however this conclusion is based on demographic descriptions of participants, with no information on who is *not *participating in the program [38].

The current study made use of a unique opportunity to link together population-based information on all births in the Canadian province of Manitoba, with prenatal and infant program participation data, to describe uptake of the program. In 2001, the Healthy Baby program was introduced province-wide in Manitoba by the Healthy Child Manitoba Office. The goal of this program was to promote prenatal and perinatal health. The Manitoba Healthy Baby program consists of two components: 1) a prenatal benefit, which is a targeted income supplement for low income women, and; 2) community support programs, which are educational and supportive groups available to all women from the prenatal period through to an infant's first birthday. The goal of this study was to determine whether groups of women targeted by the Health Baby program (e.g., low income) were participating in each of the components of the program and to identify maternal factors associated with participation in the program. We hypothesized that participation by target groups would be greater for the prenatal benefit component than the community support programs, and that not all those eligible for or targeted by the Healthy Baby program would be participating.

## Methods

### Population and Data Source

This study took place in Manitoba, a province of 1.2 million people in the geographic centre of Canada. All data came from the Manitoba Population Health Research Data Repository which houses population-based information on health and social service utilization for all residents of Manitoba. Due to comprehensive universal health care coverage, virtually all contacts with the health care system are captured [39,40]. The databases used in this study included: hospital discharge abstracts (which include up to 25 ICD10-CA diagnosis codes); physician visit records (which include an ICD-9-CM diagnosis code); the population registry (which includes demographic information on all residents registered for health care); newborn screening forms (which assess biological and social risk factors for families with a newborn, and are completed by Public Health Nurses on about 85% of all live births in the province); small-area census information (linked by residential postal codes); and social allowance management information (which includes information on all residents receiving provincial income assistance). Individual-level information from these data sources was linked across data sets using encrypted identifying numbers. The validity of the data in the Repository is well-documented [39,41-46].

The study examined all Manitoba women having a live birth in hospital from April 1, 2004 through March 31, 2008 (N = 56,560). Program data from the Healthy Baby program came from the Healthy Child Manitoba Office and included information on: 1) women receiving the prenatal benefit of up to $81 (CAD) per month during the second and third trimesters of pregnancy; 2) women participating in prenatal and/or postnatal community support programs, which varied in content across communities, but common goals included encouraging early and regular prenatal care, promoting healthy infant development, and improving nutrition. The program components are promoted through doctors' offices, posters within communities (e.g., bus shelters, community centre bulletin boards) and websites. These data were linked to birth records in the hospital abstract database to identify women participating in either component of the Healthy Baby program who had a live birth during the study period.

The study protocol was approved by the University of Manitoba Health Research Ethics Board (H2008:187), the Manitoba Health Information Privacy Committee, Manitoba Family Services and Consumer Affairs, and the Healthy Child Manitoba Office.

### Variables Used in Analyses

#### Independent variables

Several characteristics of women giving birth that are potentially related to participation in either component of the Healthy Baby program were identified as covariates: 1) mother's age at the time of the baby's birth was dichotomized as < 20 and 20+ years; 2) parity was categorized as 0, 1, 2 or 3+ previous births; 3) adequacy of prenatal care was defined using date of initiation and number of prenatal visits according to the index created by Alexander and Kotelchuck [47]; 4) region of residence was categorized as urban (Winnipeg, population = 675,000, and Brandon, population = 45,000)) and rural (the rest of Manitoba), with rural divided geographically into south and mid, and north Manitoba; 5) a composite measure of area-level SES comprising information from the 2006 Canada census on employment, education, lone-parent families and income [48,49], based on areas of approximately 400 people; 6) indication of receipt of income assistance for at least one month during pregnancy; 7) marital status; 8) high school completion; and, 9) maternal depressive symptoms. Table 1 shows the percent of women in each of the study populations with each of these characteristics.

**Table 1:** Characteristics of Study Populations

	All Women Giving Birth (N = 56560)			Women Giving Birth who received IA (N = 8183)		
**Variable**	**All (%)**	**PB (%)**	**CSP (%)**	**All (%)**	**PB (%)**	**CSP (%)**

Teen mother	8.6	16.7	11.2	19.4	18.6	20.9

Parity 0	39.0	36.7	52.1	26.4	26.9	33.9

Parity 1	31.6	24.5	25.8	26.9	26.5	24.2

Parity 2	15.5	16.5	11.8	19.9	19.7	16.3

Parity 3	13.9	22.3	10.4	26.9	26.9	25.6

adequate PNC	28.2	22.0	34.0	18.2	20.5	24.0

urban	55.0	50.8	54.9	68.3	72.6	76.3

north	11.4	16.7	3.2	12.6	9.4	5.7

Mid/south	33.6	32.5	41.9	19.1	18.1	17.9

Mean SES (high value = low SES)	0.33	1.00	0.30	1.00	1.03	0.97

on IA during pg	14.6	36.3	27.4	100	100	100

married	69.6	36.2	65.6	21.8	15.4	21.5

high school	61.0	42.8	69.1	27.8	29.6	30.9

Depressive symptoms	9.8	11.9	16.5	30.3	28.6	24.2

PB = prenatal benefit receipt

CSP = community support program participation

PNC = Prenatal Care

SES = Socioeconomic status. Composite measure based on area-level education, employment, family structure and income.

IA = income assistance.

#### Dependent variables

The two outcomes studied were receipt of the prenatal benefit and participation in community support programs. We looked at factors associated with participation in either component of the Healthy Baby program for two groups of women: 1) all women giving birth during the study period; 2) all women giving birth during the study period who received income assistance for at least one month during pregnancy.

### Analysis

The statistical method used in our analysis was Generalized Linear Models (GLM) with a binomial distribution. Several predictors (see "Variables Used in Analysis") which were believed to be associated with a mother's uptake of the prenatal benefit and/or her participation in community support programs were entered into the regression models and parameter estimates and 95% confidence intervals for each of the predictors were produced. Exponentiation of the parameter estimates allowed us to calculate the odds ratios for each of the predictors. Separate regressions were run for the two groups of women giving birth defined above. Models were run separately for prenatal benefit receipt and community support program participation (four models in total). Women living in First Nations communities, and two small communities in southern Manitoba, were excluded from analyses of community support program participation because their participation was not known. A small percentage of women participating in community support programs declined to share information on their participation, so were also removed from the analysis of support program participation. These exclusions resulted in the removal of 8553 births (15.1% of total population) from the community support program analysis. Thus, for the full population of women giving birth, there were 56560 births in the analysis of prenatal benefit receipt and 48007 births in the analysis of community support program participation. For the analysis of women receiving income assistance during pregnancy, there were 8183 births in the analysis of prenatal benefit receipt and 7398 births in the analysis of community support program participation. For regressions where the outcome was receipt of prenatal benefit, then prenatal community support program participation was entered as another covariate in the models; where community support program participation was the outcome, prenatal benefit receipt was entered into the models. All analyses were performed using SAS version 9 [50].

## Results

**Figure 1:**
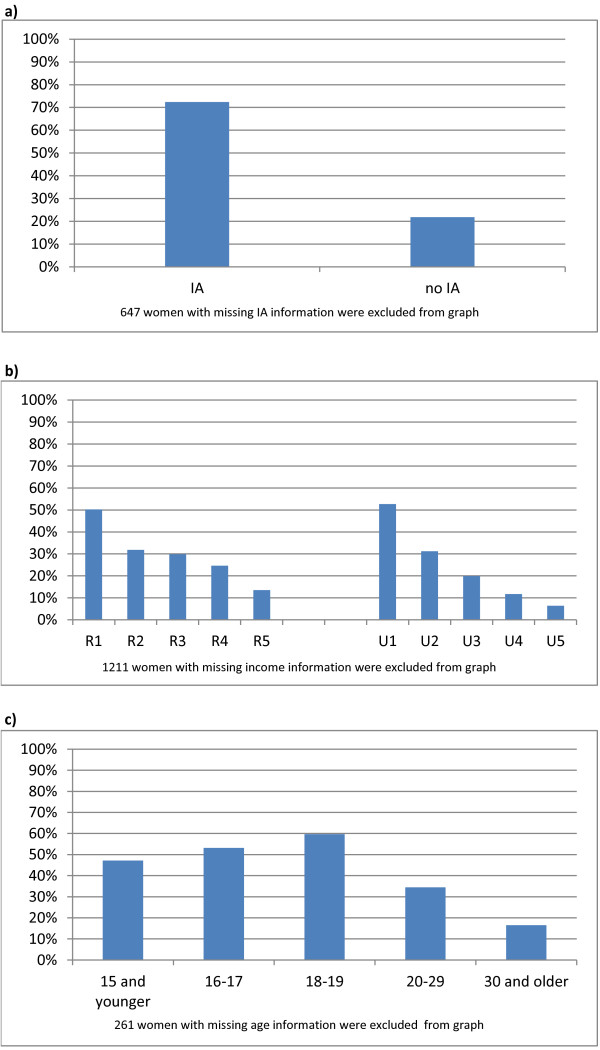
**Uptake of prenatal benefit by women at risk for poorer perinatal outcomes**. a) By receipt of income assistance. b) By area-level income. c) By mother's age at current birth.

Of the 56,560 hospital births in the study period, 16,540 (29.2%) were to women who received the Healthy Baby prenatal benefit. There was little variation in these percentages over the 4 years of the study period. Figure 1 shows the percent of births to women who received the prenatal benefit by groups that were targeted by the Healthy Baby program. Nearly three-quarters (72.4%) of women receiving income assistance during pregnancy received the prenatal benefit, compared to 21.8% of women not receiving income assistance (Figure 1a). Over 50% of women living in the lowest rural (50.2%) and urban (52.7%) income areas received the prenatal benefit, compared to 13.6% in the rural highest and 6.4% in the urban highest income areas (Figure 1b). 57.0% of mothers who were teens received the prenatal benefit, compared to 26.8% of mothers who were 20 year or older when they gave birth (Figure 1c).

**Figure 2:**
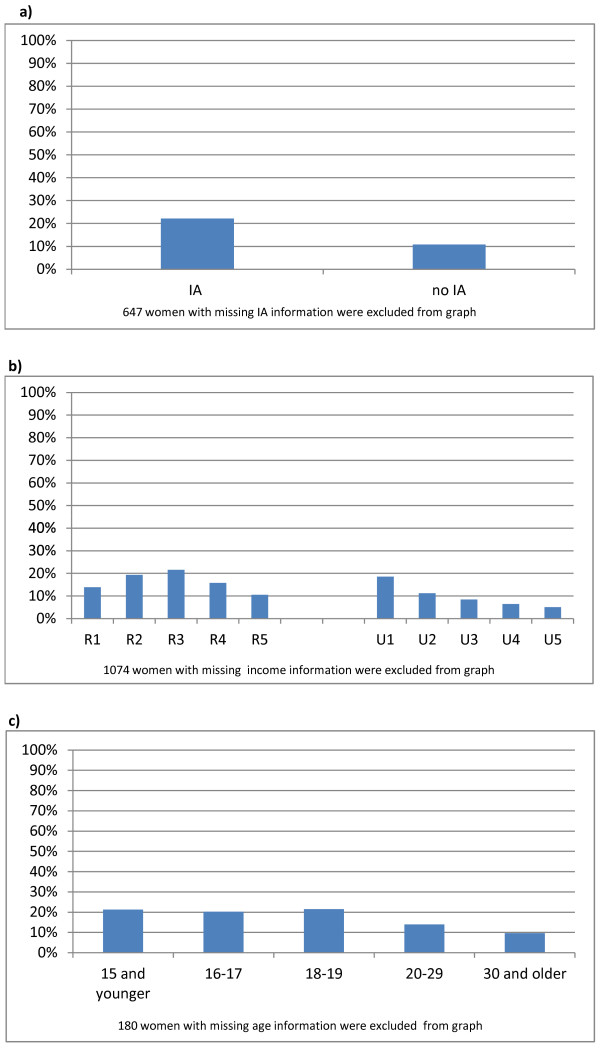
**Participation in community support programs by women at risk for poorer perinatal outcomes**. a) By receipt of income assistance. b) By area-level income. c) By mother's age at current birth.

Of the 48,007 hospital births to women living in communities where we had information on community support program participation, 6063 (12.8%) were to women who participated in at least one community support program during pregnancy and/or up to one year postnatally. Figure 2 shows the percent of births to women participating in community support programs by groups targeted by the Health Baby program. Just over one fifth (22.1%) of women receiving income assistance during pregnancy participated in any Community Support Program, compared to 10.8% of women not receiving income assistance (Figure 2a). Less than one fifth of women living in the lowest rural (12.9%) and urban (18.5%) income areas participated in community support programs (Figure 2b), compared to 10.5% and 5.1% for the highest rural and urban income areas respectively. Just over one fifth of teen mothers (21.2%) participated in community support programs (Figure 2c), compared to 12.1% of those 20 or older.

Regression analysis results identifying factors associated with receipt of the prenatal benefit and/or participation in community support programs for all women giving birth are given in Table 2.

**Table 2:** Odds Ratios (95% CI) for Factors Associated with Receipt of Prenatal Benefit and Participation in Community Support Programs, All Women Giving Birth in Manitoba, 2004/05-2007/08

Variable	PB		CSP	
	
	OR	(95% CI)	OR	(95% CI)
**Mother's Age (< 20 vs 20+)**	**1.31**	**(1.21, 1.41)**	**1.18**	**(1.06, 1.31)**
**Parity (0 vs 3+ children)**	**0.6**	**(0.56, 0.65)**	**2.04**	**(1.84, 2.26)**
**Parity (1 vs 3+ children)**	**0.52**	**(0.48, 0.56)**	**1.14**	**(1.02, 1.27)**
**Parity (2 vs 3+ children)**	**0.67**	**(0.62, 0.73)**	0.97	(0.86, 1.09)
**PNC (Adequate vs Inadequate)**	1.02	(0.96, 1.07)	**1.18**	**(1.11, 1.26)**
**Region (North vs urban)**	**0.73**	**(0.68, 0.79)**	**0.85**	**(0.73, 1.00)**
**Region (South/Mid vs urban)**	**1.17**	**(1.11, 1.23)**	**2.16**	**(2.03, 2.29)**
**SES index (higher = lower SES)**	**1.52**	**(1.49, 1.55)**	**1.24**	**(1.06, 1.29)**
**IA during pregnancy**	**3.47**	**(3.26, 3.71)**	**2.29**	**(2.09, 2.51)**
**Marital status (married vs not)**	**0.2**	**(0.19, 0.21)**	**1.09**	**(1.00, 1.09)**
**High School Completion**	**1.23**	**(1.17, 1.31)**	**1.81**	**(1.68, 1.95)**
**Maternal Depressive Symptoms**	**1.21**	**(1.12, 1.30)**	**1.49**	**(1.38, 1.62)**
**Prenatal CSP (yes vs no)**	**4.47**	**(3.93, 5.09)**	////	////
**Prenatal CSP (unknown vs no)**	**2.26**	**(1.36, 3.82)**	////	////
**Receipt of PB**	////	////	**2.10**	**(1.95, 2.26)**

Bolded values indicate statistical significance at p < 0.05

CSP = Community Support Programs

PB = Prenatal Benefit

PNC = Prenatal Care

IA = Income Assistance

For all women giving birth (total study population), maternal factors associated with increased odds of both prenatal benefit receipt and community support program participation were being a teen mother, living in south/mid rural compared to urban Manitoba, living in a low SES area, receiving income assistance during pregnancy, having completed high school, and being rated as depressed by a Public Health Nurse during the early postnatal period. Living in north Manitoba was associated with lower odds of prenatal benefit receipt and community support program participation compared to women living in urban Manitoba. For two maternal factors, the associations with prenatal benefit receipt were different than the associations with community support program participation. Having fewer children and being married were both associated with decreased odds of prenatal benefit receipt but increased odds of community support program participation. Receipt of adequate prenatal care was associated with increased odds of community support program participation, but was not associated with prenatal benefit receipt. Prenatal community support program participation was associated with increased odds of prenatal benefit receipt, and receipt of the prenatal benefit was associated with increased odds of community support program participation.

Regression analyses were repeated including only those women who received income assistance during pregnancy (Table 3). Maternal factors associated with increased odds of both prenatal benefit receipt and community support program participation for women receiving income assistance during pregnancy were receipt of adequate prenatal care, living in a lower SES area, and being rated as depressed by a Public Health Nurse during the early postnatal period. Living in northern compared to urban Manitoba was associated with reduced odds of receiving the prenatal benefit and participating in community support programs for this group of women. Being married was associated with reduced odds of receiving the prenatal benefit, but increased odds of participating in community support programs. Living in rural south/mid compared to urban Manitoba was also associated with reduced odds of prenatal benefit receipt but increased odds of community support program participation. Being a teen mom was associated with reduced odds of receiving the prenatal benefit, but was not associated with community support program participation. Number of children was not associated with prenatal benefit receipt, but having fewer children was associated with increased odds of participation in community support programs whereas having more children was associated with decreased odds. Having completed high school was associated with increased odds of receiving the prenatal benefit, but was not significantly associated with participation in community support programs. As was found for the analysis including all women giving birth, for women receiving income assistance during pregnancy participation in prenatal community support programs was associated with increased odds of prenatal benefit receipt and receipt of the prenatal benefit was associated with increased odds of community support program participation.

**Table 3:** Odds Ratios (95% CI) for Factors Associated with Receipt of Prenatal Benefit and Participation in Community Support Programs, Women Receiving Income Assistance During Pregnancy, 2004/05-2007/08

Variable	PB		CSP	
	
	OR	(95% CI)	OR	(95% CI)
**Mother's Age (< 20 vs 20+)**	**0.75**	**(0.64, 0.88)**	0.96	(0.81, 1.14)
**Parity (0 kids vs 3+ kids)**	0.99	(0.83, 1.18)	**1.44**	**(1.21, 1.72)**
**Parity (1 kid vs 3+ kids)**	0.88	(0.76, 1.02)	0.90	(0.77, 1.06)
**Parity (2 kids vs 3+ kids)**	0.89	(0.76, 1.04)	**0.79**	**(0.66, 0.94)**
**PNC (Adequate vs Inadequate)**	**1.67**	**(1.43, 1.94)**	**1.27**	**(1.11, 1.46)**
**Region (North vs urban)**	**0.4**	**(0.35, 0.47)**	**0.77**	**(0.61, 0.98)**
**Region (South/Mid vs urban)**	**0.74**	**(0.65, 0.84)**	**1.29**	**(1.1, 1.51)**
**SES (higher = lower SES)**	**1.13**	**(1.07, 1.19)**	**1.15**	**(1.08, 1.22)**
**Marital status (married vs not)**	**0.25**	**(0.22, 0.28)**	**1.27**	**(1.10, 1.47)**
**High School Completion**	**1.54**	**(1.36, 1.75)**	1.11	(0.98, 1.27)
**Maternal Depressive Symptoms (yes vs no)**	**1.17**	**(1.00, 1.36)**	**1.39**	**(1.21, 1.60)**
**Prenatal CSP (yes vs no)**	**3.35**	**(2.66, 4.22)**	////	////
**Prenatal CSP (unknown vs no)**	**3.09**	**(1.34, 7.12)**	////	////
**Receipt of HBPB**	////	////	**2.56**	**(2.18, 3.00)**

Bolded values indicate statistical significance at p < 0.05

CSP = Community Support Programs

PB = Prenatal Benefit

PNC = Prenatal Care

IA = Income Assistance

## Discussion

Close to one third of all births (29%) in Manitoba were to women who received the Healthy Baby prenatal benefit during pregnancy, and the benefit was received by a majority of the women the program was designed to reach. Almost three-quarters of women receiving income assistance during pregnancy and over half of women in low income areas received the prenatal benefit. Furthermore, over half of teen mothers received the benefit, compared to about one quarter of mothers 20 years of age and older. In contrast, less than 13% of all births in Manitoba were to women who participated in Healthy Baby community support programs, and relatively small proportions of target groups participated in these programs. Just over one-fifth of women receiving income assistance during pregnancy and the same proportion of teen-aged mothers participated in community support programs, and less than one-fifth of women from low income areas participated.

Despite the relatively higher uptake of the prenatal benefit compared to the community support programs, there is still room for improvement in prenatal benefit participation. Over one quarter of women receiving income assistance, a group all eligible to receive the benefit, did not apply to receive it, and many teen mothers and women living in low income areas also did not receive the benefit. Thus, efforts to increase enrolment by target groups in the prenatal benefit program are warranted. Furthermore, efforts to enhance participation in community support programs are required, given that a majority of the target population did not participate in these programs. The regression analyses conducted in this study identified a number of factors associated with participation in both of the components of the Healthy Baby program, providing program developers and policy-makers with important evidence of how to improve participation.

When looking at the entire population of women giving birth, we found that although women with more children were more likely to receive the prenatal benefit, they were less likely to attend community support programs. Not all Healthy Baby community support programs offer child care services for children not involved in the program (children over 1 year of age) and this may be a barrier for participation by mothers with more than one child. The financial cost and difficulties of transporting multiple children to the programs may also be a barrier. Strategies to reduce the barriers to participation in community support programs for women with multiple children should be considered.

Obtaining adequate prenatal care was associated with greater participation in community support programs for both the entire population of women giving birth and for the income assistance population, and with prenatal benefit receipt for the income assistance population. One of the ways pregnant women learn about the Healthy Baby program is through their prenatal care provider, so any efforts to increase early initiation of prenatal care will not only potentially benefit the pregnancy [51-53], but also increase the likelihood that women will find out about and participate in the Healthy Baby program. Women at risk for inadequate prenatal care are also the same groups who have lower uptake of both components of the Healthy Baby program [52,54]. Strategies that have proven effective for increasing prenatal care elsewhere, such as public health outreach to disadvantaged groups [55], should be considered in Manitoba.

For both the entire population of women and the income assistance population, unmarried women were more likely to receive the prenatal benefit, but less likely to participate in community support programs. The lower participation in support programs associated with not being married could suggest that unmarried women feel stigmatized or less welcome in the support groups. Anecdotal reports suggest that at least at some community program sites, married, middle-class mothers "take over" the program, which may make attendance for lower income, unmarried mothers less appealing. If universal access to the community support programs is to continue, strategies to make programs welcoming to women from a broad range of socio-demographic backgrounds should be explored.

For the women giving birth who were on income assistance, high school completion was associated with receipt of the prenatal benefit. The completion of the three-page application form may be an onerous task for women with limited literacy skills. Providing assistance with the application process or simplifying the application form may encourage women with lower literacy skills to apply for the program. Having automatic enrolment in the benefit program for pregnant women receiving income assistance would increase the uptake by this group [56]. Although we did not have data on immigrant status, it is likely that language was also a barrier for completion of the application for some women; translation of the form into other languages may encourage immigrant women whose first language is not English to apply for the program [57].

The finding that women with depressive symptoms, whether from the entire population or the income assistance population, were more likely to participate in community support programs and more likely to receive the prenatal benefit presents an opportunity for mental health interventions. Both prenatal and postnatal maternal depression have been implicated in impaired fetal and infant development [58-60]. For example, maternal postpartum depression has been associated with lower social engagement, more negative emotional responses and greater stress reactivity in 9-month-old infants [58]. Studies have also found that maternal depression can have long-lasting impacts on child development [61] underscoring the need for early intervention and the role the Healthy Baby program can play in identifying that need in some mothers.

The major strength of this research came from the ability to link population-based administrative data on women giving birth together with program participation data. This allowed us to determine not only who was participating in the program but also who was not participating. Together with the information on factors associated with program participation, this study provides important information to policy makers and those implementing programs for expectant and new mothers on improving uptake of programs. A limitation of the study was the lack of available data on participation in community support programs run by the Canada Prenatal Nutrition Program (CPNP). CPNP runs the community support programs in First Nations communities and two small southern rural communities. Unlike the programs run by Healthy Baby, CPNP does not collect person-level information on program participation, making it impossible not only to determine participation rates in these communities, but also to identify the factors in these communities that are associated with program participation.

## Conclusions

The Manitoba Healthy Baby prenatal benefit is reaching a reasonable proportion of women at risk for poor perinatal outcomes, however there are opportunities to improve uptake of the program. The Manitoba Healthy Baby community support programs are not reaching the majority of women at risk for poor perinatal outcomes and efforts to increase participation are required. Identifying factors associated with uptake of these two components of the Healthy Baby program can help to inform those delivering the programs of where efforts are needed to increase participation.

## List of abbreviations

CAD: Canadian dollars; ICD10-CA: International Classification of Diseases, Version 10, Canadian enhancements; SES: Socioeconomic status; UK: United Kingdom.

## Competing interests

The authors declare that they have no competing interests.

## Authors' contributions

MB developed the design for the study, with input from the other authors. Analysis of the data was carried out by WA, with direction and input from the MB and MC. Interpretation of the data involved all authors. The manuscript was drafted by MB, with significant contributions from the other authors. All authors read and approved the final manuscript.

## Authors' information

MB is a Senior Research Scientist with the Manitoba Centre for Health Policy and Associate Professor of Community Health Sciences, Faculty of Medicine, University of Manitoba. MC is a Research Scientist with the Manitoba Centre for Health Policy and Assistant Professor of Community Health Sciences, Faculty of Medicine, University of Manitoba. WA is a Data Analyst at the Manitoba Centre for Health Policy. JS is a Research Coordinator at the Manitoba Centre for Health Policy.

## Pre-publication history

The pre-publication history for this paper can be accessed here:

http://www.biomedcentral.com/1471-2458/11/691/prepub
